# Biomarkers for patients with trauma associated acute respiratory distress syndrome

**DOI:** 10.1186/s40779-017-0134-5

**Published:** 2017-08-16

**Authors:** Wujian Xu, Yong Song

**Affiliations:** 1 Department of Respiratory Medicine, Jinling Hospital, 305 Zhongshan Road, Nanjing, 210002 China; 2 Nanjing University Institute of Respiratory Medicine, Nanjing, 210002 China

**Keywords:** Trauma, Injury, Acute respiratory distress syndrome, Biomarkers

## Abstract

Trauma is a major factor that contributes to the risk for acute respiratory distress syndrome (ARDS). Biomarkers that predict the risk, diagnosis, treatment response and prognosis of ARDS after trauma have been widely investigated. In addition to their applications in clinical diagnosis and treatment, these biomarkers provide important insights into our understanding of the pathogenesis of ARDS. This review begins with a brief introduction regarding the incidence and pathogenesis of trauma-associated ARDS. Then, we focus on reviewing the clinical trials that have been designed to investigate the value of biomarkers in ARDS after trauma. Biomarkers with a confirmed value in ARDS have been organized on the basis of key pathogenic processes that are central to ARDS and are described in detail. Among these, angiopoietin 2 (Ang-2), L-selectin, Clara cell protein 16 (CC16), soluable receptor for advanced glycation end products (sRAGE), Surfactant protein D (SP-D)**,** histones, mtDNAs and some biomarker panels had a certain association with the diagnosis and prognosis of trauma-related ARDS. Further investigations are needed regarding the design of trials, the best sampling approaches and the optimal combinations of the biomarker panels.

## Background

Acute respiratory distress syndrome is a type of severe inflammatory disease of the lung that is characterized by persistent refractory hypoxemia and accompanied by opacification of chest radiographs. It was first described in 1967 among critically ill patients [1]. Although important advances have been made in the diagnosis and treatment of this disease since that time, it remains an important factor that contributes to the morbidity of critically ill patients [2].

ARDS is clinically a heterogeneous disease. The initial insults that result in ARDS vary among patients, but include sepsis, aspiration, transfusion and trauma. While sepsis is the leading risk factor for ARDS, trauma is reported to be the cause of 7% of the ARDS cases [3]. The result of an investigation conducted by the National Trauma Databank showed that the incidence of ARDS was nearly 6.5% among trauma patients who had been treated with mechanical ventilation for longer than 48 h [4]. However, patients with trauma-associated ARDS differ from cases of ARDS that were caused by other clinical factors. In particular, those with trauma-associated ARDS tend to be younger and have less acute or chronic illness [5], and the mortality of trauma-associated ARDS is estimated to be 24%, which is much lower than that for sepsis-induced ARDS [3]. This indicates that trauma-associated ARDS has its own characteristics and deserves further investigation [5].

The definition of trauma-associated ARDS is that it is ARDS caused by systemic inflammation induced by trauma to regions other than the chest. The latest definition of ARDS requires the presence of acute onset of hypoxemia, a ratio of the partial pressure of arterial oxygen (PaO_2_) in mmHg to the fraction of inspired oxygen (FiO_2_) of less than 300, bilateral infiltrates on chest radiograph, and a lack of evidence of cardiac failure or fluid overload [6]. These updated criteria, which were released in 2012, are mainly based on clinical and radiographic outcomes and are valuable for the assessments of severity and prognosis. In the context of trauma-associated ARDS, the current criteria are less applicable because there is enormous heterogeneity among the patients with injury [7]. Recently, intensive efforts have been made to identify indicators that are associated with the pathological processes of the disease. A series of biomarkers for trauma-associated ARDS have been widely investigated in these patients and showed a different pattern from those for other ARDS patients. This review focuses on the biomarkers in trauma-associated ARDS patients that can provide new insights in predicting the risks, assessing the disease severity and the evaluating the prognosis.

## Pathogenesis of ARDS

ARDS is a progressive inflammatory disease of the lungs, which can generally be divided into the acute, proliferative and fibrotic phases according to ultrastructural details at different time points in the disease [8]. The acute phase is characterized by a prolonged, uncontrolled inflammatory response in the lung tissues. There is a significant accumulation of neutrophils, macrophages and other inflammatory cells in the alveoli. Diffuse alveolar damage is the hallmark pathological change of ARDS, although it appears in only half of the patients [9, 10]. It is characterized by epithelial barrier and endothelial dysfunction followed by disturbances in the coagulation and fibrolysis systems. The injured epithelial and endothelial cells allow flooding of proteinaceous fluid and cellular debris into the alveoli and the formation of hyaline membranes. In the proliferative phase, the remaining edema is gradually reabsorbed, and the infiltrated neutrophils are removed and replaced with mononuclear cells and alveolar macrophages. Type II alveolar epithelial cells become hyperplastic in an attempt to repair the epithelial barrier. As the phase progresses, granulation tissue develops in the alveolar spaces and septae, which leads to organizing fibrosis. In the fibrotic phase, there is evidence for collagen deposition and fibrosis of the alveolar walls, which results in an irreversible impairment of lung function. However, in many patients, only resolution of the edema and acute inflammation are manifested without evidence of fibrosis [11].

## Biomarkers in ARDS

Numerous biomarkers have been tested for the diagnosis and treatment of ARDS [12]. In several trials conducted by the National Heart Lung and Blood Institute’s ARDS Clinical Trials Network (Ardent), including the conventional versus low tidal volume ventilation trail (ARMA) [13] and the low versus high end-expiratory pressure in ARDS trail (ALVEOLI) [14], the investigators examined the potential roles of serum biomarkers in ARDS patients. They found that a panel of biomarkers, including Ang-2, Von Willebrand factor (vWF) and others, could be used as predictors for mortality and prognosis in ARDS patients. These biomarkers, even though some of them were found not to be associated with the clinical prognosis, can be used as markers for understanding the key cellular injury pathways central to lung injury: the inflammatory cascade, epithelial/endothelial injury, disordered coagulation and fibrolysis, and fibrosis.

The etiology of trauma may include thermal injury, inspiration of notorious smoke or fluid, or release of damage-associated molecular pattern molecules that result from blunt tissue injury. As a result, trauma-associated ARDS is clinically and biologically different from ARDS caused by other disorders [5]. Some biomarkers in patients with other types of ARDS have also been tested in trauma-associated ARDS. Some trauma-specific biomarkers such as mitochondrial DNA have also been investigated in these ARDS patients [15]. The results of these studies showed that the biomarkers in trauma-associated ARDS have unique characteristics, which we discuss in detail as follows (Table 1).

**Table 1:** Verified biomarkers for the diagnosis and prognosis of trauma-associated acute respiratory distress syndrome

Injury pathway	Biomarker	Pattern in ARDS	Clinical association	Statistic description	Reference
Endothelial injury	Ang-2	elevated	Predicting ARDS mortality	AUC: 0.74 (95% CI, 0.62–0.84) OR: 4.0 (95% CI, 1.6–10.2)	[18]
					[19]
	L-Selectin	decreased	Higher ARDS incidence	Serum Concentration in ARDS: 262.7 g/L (95% CI, 113.5–411.9 g/L)	[23]
Epithelial injury	sRAGE	elevated	Early onset of ARDS ARDS incidence	1773 (949–3227) vs. 1226 (773–1944) Fold change: 3	[26]
					[27]
	SP-D	decreased	Higher mortality Worse oxygenation ARDS diagnosis	AUC: 0.69 (95% CI, 0.6, 0.76)	[29]
					[29]
					[30]
	CC16	elevated	Lung contusion volume ARDS mortality	*r* = 0.78, *P* < 0.0001	[31]
					[33]
Coagulation	Histones	elevated	ARDS incidence SOFA scores	Fold change: 6.72 *r* = 0.55, *P* < 0.01	[40]
					[40]
Inflammation	mtDNA	elevated	SIRS mortality	AUC: 0.725 (95% CI 0.613–0.837). RR: 20.4 (95% CI, 1.3–318)	[43]
					[45]

*Ang-2* Angiopoietin-2, *ARDS* Acute respiratory distress syndrome, *AUC* area under the curve, *CC16* Clara cell protein 16, *mtDNA* Mitochondrial DNAs, *OR* Odds ratio, *RR* Relative risks, *SIRS* Systemic inflammatory distress syndrome, *sRAGE* Soluble receptor for advanced glycation end products, *SP-D* surfactant protein D, *WMD* weighted mean difference

## Biomarkers of pathogenesis

### Endothelial biomarkers

Vascular leakage plays a key role in ARDS. Increased vascular permeability is an early event in the initial stage of ARDS. The activated inflammatory cells as well as the mediators they release are harmful to the endothelial cells and result in increased expression of cell surface molecules and expanded intercellular spaces, which are beneficial for leukocyte accumulation and transmigration. As a result, protein-rich fluids and activated inflammatory cells from the blood accumulate in the pulmonary alveoli. Various biomarkers have been examined in plasma and bronchial alveolar lavage fluid (BALF) in ARDS patients, including Ang-2, ICAM-1, selectins, VEGF and others.

#### Ang

Ang is a member of the vascular growth factor family, whose signaling is associated with angiogenesis. Ang-1 and Ang-2 are the most completely described members of the angiopoietin family. Ang-1 plays important roles in vessel maturation, adhesion, migration and survival, whereas Ang-2 is critical in vascularization. During inflammation, Ang-1 helps to stabilize the endothelial cells and inhibit leukocyte adhesion and thus reduces vascular permeability [16]. On the other hand, Ang-2, a natural antagonist to Ang-1 that is released by activated endothelial cells, increases the vascular permeability and sensitizes endothelial cells to inflammatory stimuli [17].

In ARDS, the plasma Ang-1 and Ang-2 levels increase during the development of the disease [18]. In the fluid- and catheter-treatment trial conducted by the Ardent, Calfee et al. measured the plasma levels of Ang-2 in 931 patients and found that the baseline Ang-2 levels were associated with ARDS mortality [19]. Furthermore, the concentration of this mediator was reduced after conservative fluid treatment. To explore the value of angiopoietin in trauma-associated ARDS, Ganter MT et al. measured the plasma levels of Ang-1 and Ang-2 soon after trauma [20]. They found that in 208 adult trauma patients, the plasma levels of Ang-2 but not Ang-1were increased and were correlated with the severity of the injury and tissue hypoperfusion. Moreover, the Ang-2 levels correlated with markers of endothelial activation and were associated with a worse clinical outcome. The patients with higher Ang-2 levels had a higher mortality rate and an increased incidence of acute lung injury [20]. Another study revealed that the plasma Ang-2 levels were regulated by agenetic variant of ANGPT2. It was found that two SNPs in ANGPT2 (rs1868554 and rs2442598) were significantly associated with higher Ang-2 levels and acute lung injury (ALI) morbidity in trauma patients [21]. These results suggest that Ang-2, as a marker of vascular permeability, is useful in predicting ARDS susceptibility in trauma patients.

#### Selectins

The selectins are a family of cell adhesion molecules. There are three known members of the selectin family, named L-, E- and P-selectin, which are presented on activated leukocytes, endothelial cells and platelets, respectively. Elevated E- and P-selectin have been found among ARDS patients [22, 23], whereas L-selectin shows a different pattern in response to inflammation. In a recent meta-analysis, 7 prospective studies involving 350 patients were included in an analysis of the value of the soluble adhesion molecule L-selectin in predicting major complications after severe trauma. Lower plasma L-selectin concentrations were found among patients who progressed to ALI/ARDS. Non-survivors had significantly lower levels of early plasma L-selectin than survivors. This result indicates that early decreases in plasma L-selectin may predict an increased morbidity of ALI/ARDS in trauma patients [24].

### Epithelial biomarkers

Human alveoli are lined with two types of epithelial cells, designated the type I and type II alveolar cells. The type I alveolar epithelial cells are long, thin cells that comprise the wall of the alveolus. They form the main parts of the capillary-alveolar barrier and are responsible for fluid reabsorption from the alveoli. The type II alveolar epithelial cells are cube-like cells located between the type I cells and play critical roles in maintaining the surface tension of alveolus and repairing the injured lung tissues in response to inflammation. In addition to these two types of cells, another important type of epithelial cell, the Clara cells, participate in the process of ARDS, despite the limited numbers of these cells. During acute injury, specific proteins from these epithelial cells could be released into blood and the alveolar fluid, and could be used as biomarkers to assess the severity of the alveolar epithelial injury.

#### Soluble receptor for advanced glycation end products (sRAGE)

RAGE is a member of the immunoglobulin superfamily that is responsible for infection, injury and inflammation. It is highly expressed in type I alveolar epithelial cells. The soluble receptor of RAGE (sRAGE) is a competitive decoy receptor for membrane RAGE and can be detected in blood and BALF. It has therefore been used as a marker of alveolar epithelial type I cell injury [25]. sRAGE levels are elevated in ARDS patients and are associated with other physiologic markers of more severe disease [26]. For trauma-associated ARDS, patients with an early onset of ARDS on day 1 or 2 had the highest plasma levels of sRAGE [27]. Consistent with this result, a recent investigation reported that among 130 patients with multiple traumas, the plasma sRAGE levels were almost 3 times higher than a matched set of healthy controls, but these levels decreased by 41% on day 2. Higher initial sRAGE levels were found in the patients who developed lung injury. Moreover, there was a significant correlation between the initial sRAGE levels and the relative lung contusion volume [28]. These results suggest sRAGE levels measured shortly after trauma could be used as a tool to diagnosis and predict the early onset of ARDS.

#### Surfactant protein D (SP-D)

Pulmonary surfactant proteins are specifically secreted by the type II alveolar epithelial cells [29]. Of the four family members (SP-A through SP-D), the hydrophilic SP-D has been the most widely investigated in ARDS. Lower SP-D levels in the pulmonary edema fluid were found in ARDS patients with worse oxygenation, and the reduced levels were associated with an increased mortality [30]. In a clinical trial involving 200 patients with severe sepsis who were at high risk for ARDS, SP-D was found to be one of the best biomarkers for the diagnosis of ARDS [31]. Another analysis of 1451 ADRS patients revealed that trauma patients had significantly lower plasma levels of SP-D [5]. However, there is conflicting evidence regarding the use of SP-D in trauma-associated ARDS. The plasma levels of SP-D were not associated with the presence of ARDS or the extent of lung contusion among patients with multiple injuries [32]. These intriguing results indicate that more studies are needed to further understand the value of SP-D in trauma-associated ARDS.

#### Clara cell protein 16 (CC16)

CC16 is secreted by Clara cells and protects lung tissues from inflammation, oxidative stress and fibrosis in ARDS [33]. Both serum and BALF CC16 levels have been investigated in ARDS. Jorens PG et al. [34] found that the CC16 levels were elevated in both the serum and BALF among ARDS patients, and higher levels were associated with increased mortality. Similarly, in trauma-associated ARDS, Wutzler S et al. found that the serum levels of CC16 were significantly in the trauma patients who developed severe lung injury compared with non-ARDS patients and healthy controls. The CC16 levels were also found to be correlated with the volume of the lung contusions [32]. However, conflicting evidence showed that the CC16 levels in the plasma and pulmonary fluid edema were lower in ARDS patients than in patients with cardiogenic pulmonary edema [35]. Regardless of etiology of ARDS, the timing of sampling seems to be an important factor that might influence the CC16 concentrations. In a recent study, the investigators collected an extensive series of blood samples from the time the patients were admitted until 14 days after trauma. They found that the initial CC16 levels were significantly elevated, but if no secondary respiratory complications occurred, the levels declined to the control values within the first day after trauma [36]. These results suggest that elevated CC16 is a specific biomarker for traumatic ARDS, but the timing of sampling need further optimization.

#### Coagulation and fibrinolysis

During the development of ARDS, injuries to the endothelial and alveolar epithelial cells cause the release of various pro-coagulant mediators, which then activate the coagulation cascade. Coupled with this process, the fibrinolytic system is activated but with markedly decreased fibrinolytic activities. Consequently, microthromboli form in the pulmonary vessels, and fibrin deposits in the alveoli. Several key factors involving the coagulant and fibrinolytic system have been found to be dysregulated in ARDS. For example, lower protein C and higher plasminogen activator inhibitor-1 levels were linked to higher mortality and worse clinical outcome [37]. Elevated plasma thrombomodulin levels were also found to be associated with increased mortality in ARDS [38]. Unfortunately, none of these direct mediatorshas been tested in trauma-associated ARDS, although it is an ideal disease model to study the mechanisms of coagulant and fibrinolytic dysfunction in ARDS. Only circulating histones, a newly identified DAMP, were reported to have a correlation with the coagulation system in ARDS.

#### Histones and protein C

Histones have recently been recognized as a new kind of DAMP in ARDS [39]. Extracellular histones, together with free DNA and granular proteins, form structures called neutrophil extracellular traps, which act as a potent pro-inflammation factor during ALI/ARDS. Extracellular histones that appear in the plasma have the ability to active pulmonary endothelial cells and neutrophils in ARDS [40]. Simon TA et al. investigated the levels of histones in 52 patients with severe non-thoracic blunt trauma and found that the levels of the circulating histones increased immediately after the trauma. The histone levels were significantly associated with the incidence of ARDS, as were markers that reflected endothelial damage and coagulation activation [41]. In another predictive study, the plasma histone levels were measured in 132 severely injured trauma patients at the time of admission and 6 h later. The study revealed that plasma histones were elevated on arrival but declined by 6 h after admission. The histone levels correlated with the injury severity score, the incidence of acute lung injury and mortality. Moreover, higher histone levels also correlated with prolonged international normalized ratio (INR), partial thromboplastin time (PTT), the fibrinolytic markers D-dimer and tissue-type plasminogen activator, and activated protein C. These results indicated dysregulated coagulant and fibrinolytic systems [42]. These findings suggest that circulating histones may be used as an early predictor of ARDS and an indicator for deregulated fibrinolysis and activation of anticoagulants.

### Inflammation

An uncontrolled inflammatory cascade is the direct cause of lung injury after major trauma. Various inflammatory mediators, both pro-inflammatory and anti-inflammatory, have been measured in the plasma, serum, bronchoalveolar lavage fluid (BALF) or undiluted pulmonary edema fluid. To date, the utility of single inflammatory mediator in the diagnosis or treatment of ARDS seems limited [12]. It has therefore been suggested that a combination of these mediators is more practicable, which we will discuss in detail later. Unlike other etiologies, some specific mediators, such as the mitochondrial DNA released by the damaged tissues in trauma patients, could be used as markers to assess the risk and severity of ARDS in these patients.

#### Mitochondrial DNAs (mtDNA)

Human muscle and liver tissues are rich in mitochondria. Following major trauma, mitochondrial DNA can be released into the circulation. The plasma mtDNA levels were significantly elevated in trauma patients with an injury severity score higher than 25 [15]. These mtDNAs have recently been identified as a type of endogenous damage-associated molecular pattern that has important immune consequence. Because of this, the elevated mtDNAs are associated with systemic inflammation after trauma [43]. Our own study found that elevated plasma mtDNA could be used an independent predictor for a systemic post-traumatic inflammatory response [44]. Compared to sepsis patients, the plasma mtDNA levels peaked on the day of admission in patients with trauma [45]. This early increase in the mtDNA levels was associated with the evolution of MODS including ALI/ARDS and mortality [46].

#### Biomarker panels including inflammatory mediators

As described above, it is not surprising that single inflammatory mediator is not enough to reflect the activation of inflammatory cascade due to the complexity of ARDS. As a result, great efforts have been made to evaluate combinations of mediators for their utility in assessing ARDS.

In the VALID study, the investigators found that a panel of five biomarkers (IL-6, IL-8, SP-D, RAGE, and CC16) yielded a diagnosis accuracy of 0.78 (AUC), much higher than that of any single biomarker [31]. The largest trial using this strategy was conducted by Richard DF and his colleagues in 2011 [47]. In their retrospective nested case–control trial, a panel of 21 plasma biomarkers were measured in 192 trauma patients, among whom 107 were diagnosed with ARDS. These biomarkers include those reflecting inflammation (IL-1β, IL-2, IL-4, IL-5, IL-6, IL-8, IL-10, IL-12p70, GM-CSF, TNF-α, MPO and INF-γ), epithelial/endothelial injury (SP-D, CC16, sRAGE, Ang-2, vWF, sICAM-1, BNP), coagulant/fibrinolysis (PAI-1), and fibrosis (PCP III). Using a multivariate logistic regression model, a combination of 7 biomarkers (IL-8, IL-10, TNF-α, Ang-2, BNP, sRAGE and PCP III) was shown to have a high diagnostic accuracy in differentiating trauma patients with ARDS from those without [47]. Another reanalysis of 1451 ARDS patients revealed that a combination of TNFR-1, vWF, ICAM-1, and SP-D could discriminate the trauma patients from non-trauma patients [5]. Taken together, these results suggest that a biomarker panel has more potential application for the diagnosis and prognosis of ARDS than a single biomarker.

### Other biomarkers for predicting disease risk and outcome

In addition to the markers reviewed above, numerous other biomarkers have been tested in non-traumatic ARDS, but their utility in trauma-associated ARDS remains unclear. For example, lower plasma vWF is a good predictor for a worse mortality and clinical outcome in pooled ARDS cases, but its application in trauma-associated ARDS has not been tested. Some new biomarkers, such as SNPs of the PPFIA1 and FAAH genes, although may not play a direct role in the pathological process of ARDS, were recently found to be associated with an increased risk of ARDS after major trauma [48, 49]. As the development of next generation sequencing, metabolomics and proteomics progresses, increasing numbers of differentially expressed genes, metabolites and proteins will be identified. These markers deserve further verification of their value in the diagnosis and prognosis of trauma-associated ARDS.

## Conclusions

Trauma-associated ARDS is quite different from non-traumatic ARDS. Ang-2, L-selectin, CC16, histones and mtDNAs have been proved to have diagnostic or prognostic values for trauma-associated ARDS. These markers also provide useful information on the pathological status of the patients. However, several limitations remain in the attempt to define the best biomarker for trauma-associated ARDS. First, ‘gold standards’ are needed when designing a new trial to assess the value of biomarkers for ARDS. Some key criteria including ARDS incidence, organ failure and mortality must be included in future new trial because these factors give important information on the assessment of disease sensitivity and severity. Second, for a specific biomarker, the best way to collect an ideal biological sample should be verified. For example, several studies have reported that bronchoscopic microsampling yields a much higher concentration of inflammatory mediators than traditional bronchoalveolar lavage (BAL) methods [50]. Third, panels of biomarkers seem to be a more practical choice than a single marker. However, the best combination and calculation model deserves further verification in all patients with ARDS with different causes. Furthermore, in addition to the biomarkers, the combination of biomarkers with clinical markers should also be tested [51, 52], because this might provide a more correct insight into the development of trauma-associated ARDS.

## Abbreviations

ALI: Acute lung injury
ANG: Angiopoietin
ARDS: Acute respiratory distress syndrome
AUC: Area under curve
CC16: Clara cell protein 16
MODS: Multiple organ dysfunction syndrome
mtDNA: mitochondrial DNAs
SP-D: Surfactant protein D
sRAGE: soluble receptor for advanced glycation end-products
